# Prevalence, Serotypes, and Antimicrobial Resistance of *Salmonella* Species in Ready-to-Eat Foods in Erbil, Iraq

**DOI:** 10.3390/microorganisms13102225

**Published:** 2025-09-23

**Authors:** Dhary Alewy Almashhadany, Abdulwahed Ahmed Hassan, Izhar U. H. Khan

**Affiliations:** 1 Department of Medical Laboratory Science, College of Science, Knowledge University, Erbil 44001, Iraq; dhary1951@gmail.com; 2 Medical Laboratories Department, Alnoor University, Mosul 41002, Iraq; a.a.hassan@alnoor.edu.iq; 3 Department of Veterinary Public Health (DVPH), College of Veterinary Medicine, University of Mosul, Mosul 41001, Iraq; 4 Agriculture and Agri-Food Canada, Ottawa Research and Development Centre, 960 Carling Ave, Ottawa, ON K1A 0C6, Canada

**Keywords:** *Salmonella*, ready-to-eat foods, street-vended food, antimicrobial resistance, serotyping

## Abstract

Ready-to-eat (RTE) foods including sandwiches, pastries, shawarma, and burgers are widely consumed and may potentially increase the risk of foodborne infections. This study investigated the prevalence, serovar diversity, and antimicrobial resistance (AMR) of *Salmonella* spp. in RTE foods collected between January and June 2024 from street vendors and restaurants across Erbil, Iraq. A total of 350, including 85 sandwiches, 75 pastries, 95 shawarma, and 95 burgers obtained from 115 cafeteria, 120 street vendors, and 115 restaurants were analyzed. *Salmonella* was detected in 7.1% (n = 25) of samples, with a high contamination in shawarma (8.4%; n = 95), followed by sandwiches (7.1%; n = 85), pastries (6.7%; n = 75), and burgers (6.3%; n = 95). Street vendors exhibited a higher (9.2%; n = 120) contamination rate compared to the cafeteria (6.9%; n = 115) and restaurants (5.2%; n = 115). Among 25 *Salmonella* isolates, 10 serotypes were identified, with *S.* Anatum (20%) and *S*. Typhimurium (16%) being the most prevalent. All isolates were susceptible to colistin, cefadroxil, and gentamicin, while showing high resistance to streptomycin (52%) and levofloxacin (48%). Contamination peaked during the warmer months, particularly in June (15.4%) and May (11.5%), when compared to the other sampling months. These findings highlight significant food safety concerns related to *Salmonella* contamination and AMR in RTE foods, emphasizing the urgent need for enhanced hygiene practices and regulatory oversight especially among street vendors.

## 1. Introduction

Foodborne transmission accounts for an estimated 94% of salmonellosis cases globally. In addition to causing acute gastroenteritis, *Salmonella* spp. are also responsible for enteric (typhoid) fever, a serious systemic illness common in low-income countries [1].

Ready-to-eat (RTE) foods are those that are consumed without any additional cooking or processing prior to consumption. While convenient, these foods pose a substantial public health risk due to the absence of a final heat treatment step, which would otherwise eliminate pathogenic microorganisms. Among these pathogens, *Salmonella* spp. remain one of the most significant foodborne pathogens, capable of causing severe gastrointestinal illness, systemic infections, and in some cases, life-threatening complications [1,2].

Numerous studies have reported the presence of *Salmonella* in RTE meat, poultry, and egg-based products, even after using various cooking methods such as grilling, frying, or baking. Cross-contamination during preparation, inadequate cooking, use of contaminated ingredients, and poor hygiene practices are key contributing factors [1,2,3,4]. Non-typhoidal *Salmonella* (NTS) serovars such as *S*. Enteritidis, *S*. Typhimurium, *S*. Newport, *S*. Heidelberg, and *S*. Kentucky are strongly associated with RTE foods and have been identified as leading causes of foodborne gastroenteritis worldwide, making them highly relevant for food safety regulations, outbreak monitoring, and antimicrobial resistance surveillance [5]. Artisanal and street-vended meat products, particularly sausages, shawarma, meat pies, and burgers are vulnerable due to informal processing and lack of compliance with food safety standards. In developing countries, RTE foods sold by street vendors are widely consumed but are often prepared under unsanitary conditions, with little or no regulatory oversight [2,4].

Although studies in Iraq have reported the prevalence of *Salmonella* in local foods and grilled chicken meat [6,7], most research has been focused on raw products, such as chicken meat and eggshells [8,9]. However, these studies can help in predicting outbreak timing and target high-risk populations. Similarly, the prevalence of *Salmonella* in RTE foods varies significantly across geographic regions and seasons, reflecting differences in climate, food processing technologies, regulatory enforcement, and consumer habits. Warmer months typically show increased contamination due to favorable conditions for bacterial proliferation [10,11]. Several reports from Africa, the Middle East, Asia, and Latin America have documented *Salmonella* contamination rates in RTE foods ranging from 5% to over 40%, depending on the type of food, handling practices, and sampling environment [12,13,14]. In Iran, Egypt, Nigeria, and Brazil, RTE meat and poultry products have repeatedly been flagged as reservoirs for *Salmonella* contamination [15,16,17,18]. Studies from Ethiopia and Pakistan also revealed contamination of RTE sandwiches, pastries, and meat dishes with multidrug-resistant *Salmonella* strains, suggesting a global trend of rising public health threats from these foods [19,20].

Globalization has further exacerbated the challenge, as RTE food products are often distributed internationally, increasing the risk of cross-border transmission of pathogens. Outbreaks of multidrug-resistant (MDR) *S*. Typhimurium linked to imported RTE meats and salads have been reported in Europe and North America, underlining the need for harmonized international food safety standards and surveillance systems [21,22].

Controlling *Salmonella* remains difficult due to its ability to form biofilms and resist many common disinfectants [23]. The growing demand for minimally processed, preservative-free, and organic RTE foods adds complexity to microbial risk management, as such products may lack essential antimicrobial barriers. Chicken meat, eggs, and their derivatives are consistently among the top contributors to salmonellosis outbreaks in developing regions. Inadequate storage, substandard kitchen hygiene, and cultural practices such as consuming raw or undercooked foods further elevate the risk of infection [24,25,26].

Another critical concern is antimicrobial resistance (AMR) in *Salmonella*, which has been increasingly reported in isolates from both food and clinical settings. Resistance to fluoroquinolones, tetracyclines, and macrolides, including erythromycin, has been observed globally [27,28,29]. The routine and often unregulated use of antibiotics in poultry production, especially in broiler operations, is a significant driver of AMR development [25,26,27,28,29,30]. Resistant *Salmonella* strains not only complicate treatment but also pose a severe threat to public health by limiting therapeutic options and increasing the likelihood of outbreak persistence and spread.

Accordingly, this study was designed to investigate the prevalence, serotyping and antimicrobial resistance profiles of *Salmonella* spp. in RTE foods, including sandwiches, pastries, shawarma, and burgers, from various street vendors and restaurants in the Erbil Governorate, Iraq.

## 2. Materials and Methods

### 2.1. Food Description and Sample Collection

A total of 350 ready-to-eat (RTE) food samples including 85 roast beef sandwiches (comprising sliced beef and raw vegetables), 75 pastries (filled with minced beef and leafy greens), 95 shawarma (typically prepared from seasoned blend of lamb and beef with spices), and 95 spiced burger (containing ground beef and assorted spices), each weighing approximately 200–250 g, were collected between January and June 2024 from various locations across Erbil Governorate, Iraq. Only one sample was collected per site from randomly selected 115 cafeteria, 120 street food vendors, and 115 restaurants. All samples were aseptically collected in sterilized polyethylene bags and transported in cooled containers to maintain the cold chain. Upon arrival at the Microbiology Laboratory, College of Science, Knowledge University in Erbil, the samples were stored at 4 °C and processed for microbiological analysis within 48 h of collection.

### 2.2. Isolation of Salmonella *spp.*

The isolation of *Salmonella* spp. was carried out following ISO 6579:2002/AMD 1:2007 method, with minor modifications, including pre-enrichment in buffered peptone water (BPW), selective enrichment in Selenite broth (SB), plating on Salmonella-Shigella (SS) selective media, and presumptive colony selection [31]. Briefly, from each sample, 25 g of edible meat portions (excluding bread but including vegetables) was aseptically chopped using sterile scissors and forceps and transferred into a sterile polyethylene bag. The samples were homogenized in 225 mL of sterile 0.1% Buffered Peptone Water (BPW) for pre-enrichment, as recommended [32]. The homogenates were incubated at 37 °C for 18 ± 2 h. Subsequently, 1 mL of the incubated pre-enrichment broth was transferred to 10 mL of SB, vortexed, and incubated at 41.5 °C ± 1 for 24 h. After selective enrichment, a loopful of the SB was streaked onto SS agar plates and incubated at 37 °C for 24 h. Putative *Salmonella* colonies (typically pale with black centers) were selected for further analysis [32,33].

### 2.3. Identification of Salmonella *spp.*

Putative *Salmonella* colonies obtained from SS agar were sub-cultured onto MacConkey agar and incubated at 37 °C for 24 h. Non-lactose fermenting colonies (light or colorless, 1–3 mm, circular, smooth, and convex) were selected for microscopic and biochemical identification. Gram staining was performed to confirm the morphology of the *Salmonella* strains. Biochemical tests including Triple Sugar Iron (TSI) agar test, urease test, Sulfide-Indole-Motility (SIM), Potassium Cyanide (KCN) tolerance test, Simmons citrate utilization test, were performed to confirm the presence of *Salmonella* spp. These procedures were carried out using previously described standard protocols [7,33].

### 2.4. Serotyping

All biochemically confirmed *Salmonella* strains were subjected to serotyping using the slide agglutination method with the Remel^®^ *Salmonella* Antisera Kit (Remel Europe Ltd., Kent, UK), following the manufacturer’s instructions and standard laboratory protocols.

### 2.5. Antibiotic Susceptibility Testing

Antibiotic susceptibility of the *Salmonella* strains was assessed using the standard disk diffusion method on Mueller-Hinton agar (Oxoid, Hampshire, UK) following the modified Kirby-Bauer technique. The selection of antibiotics and their concentrations, along with the inhibition zone diameters, were interpreted according to the Clinical and Laboratory Standards Institute (CLSI) guidelines [34].

The antibiotics, including colistin (CST, 10 µg), erythromycin (ERY, 15 µg), cefadroxil (CFR, 30 µg), cefotaxime (CTX, 30 µg), neomycin (NEO, 30 µg), gentamicin (GM, 15 µg), norfloxacin (NOR, 10 µg), kanamycin (KAN, 30 µg), levofloxacin (LEV, 5 µg), streptomycin (STR, 10 µg), tetracycline (TEC, 30 µg) and tobramycin (TM, 10 µg) were tested. The procedures and interpretation were performed in accordance with established laboratory quality control standards [7].

### 2.6. Statistical Analysis

The chi-square test of independence was applied to assess the statistical significant differences in contamination rates across food types (sandwiches, pastries, shawarma, burgers) and sample sources (cafeteria, street vendors, restaurants), with a significance level of *p* < 0.05. Additionally, 95% confidence intervals (CI) were calculated for proportions using the normal approximation method to evaluate the precision of contamination estimates.

## 3. Results

### 3.1. Prevalence of Salmonella *spp.*

Of the total 350 ready-to-eat (RTE) food samples analyzed, the overall prevalence of *Salmonella* spp. was relatively low 7.1% (n = 25). However, among different food types, shawarma samples exhibited a high (8.4%; n = 95) contamination rate as compared to sandwiches (7.1%; n = 85), pastries (6.7%; n = 75), and burgers (6.3%; n = 95). Regarding the source of sample collection, a high (9.2%; n = 120) contamination rate was observed in samples obtained from street food vendors compared to cafeteria (6.9%; n = 115), and restaurants (5.2%; n = 115). A summary of positive samples categorized by both food type and sampling location is presented in Table 1.

Statistical analysis using the chi-square test yielded a *p*-value of 0.927, indicating no statistically significant association between food type or sampling source and the prevalence of *Salmonella* spp. The wide 95% confidence intervals reflect limited sample sizes in subgroups. No subgroup demonstrated a significantly higher contamination rate than others. Table 2 presents the contamination rates along with 95% confidence intervals.

### 3.2. Salmonella Serovars

Serotyping of all *Salmonella* isolates revealed 10 distinct serovars, indicating considerable diversity. The most frequently isolated serovar was *S.* Anatum (n = 5; 20%), followed by *S.* Typhimurium (n = 4; 16%). Both *S.* Newport and *S.* Dublin were identified in 3 isolates each (12%). Other detected serovars included *S.* Arizonae, *S.* Enteritidis, *S.* Infantis, and *S.* Muenchen, each with 2 isolates (8%), whereas *S.* Montevideo and *S.* Senftenberg were identified in one isolate each (4%). This distribution highlights the genetic heterogeneity of *Salmonella* strains in RTE foods.

### 3.3. Antimicrobial Sensitivity Pattern

All *Salmonella* strains were tested against a panel of 12 antibiotics. The isolates exhibited 100% sensitivity to colistin (CST), cefadroxil (CFR), and gentamicin (GM), indicating strong effectiveness of these antibiotics. Cefotaxime (CTX) also showed high efficacy, with 92% sensitivity. However, substantial resistance was observed against streptomycin (52%) and levofloxacin (48%), raising concerns about emerging resistance. Moderate resistance was noted for tetracycline (36%) and erythromycin (15%), norfloxacin (32%), and kanamycin (32%) as presented in Table 3.

### 3.4. Temporal Distribution of Salmonella Prevalence

Monthly analysis revealed seasonal variation in rate of prevalence of *Salmonella*. The positive samples were significantly (*p* < 0.05) higher in June (15.4%) and May (11.5%) than in the rest of the sampling months. The lowest prevalence was recorded in January (1.6%) and February (1.7%), indicating a potential seasonal pattern (Table 4).

## 4. Discussion

The present study provides regionally significant insights into the prevalence, serotype diversity, and antimicrobial resistance of Salmonella spp. in ready-to-eat (RTE) foods in Erbil Governorate, Iraq. It represents a comprehensive investigation in northern Iraq, evaluating seasonal variation, food-type and source-specific contamination rates, and serovar diversity in RTE foods. The findings also underscore the alarming resistance of isolates to commonly used antibiotics such as streptomycin and levofloxacin, which raises important public health concerns. These results contribute to the essential global surveillance of foodborne pathogens and antibiotic resistance data, particularly in under-reported regions. Since *Salmonella* was detected in 7.1% of the RTE food samples, this indicates a noteworthy level of contamination. Among the food categories analyzed, shawarma showed a higher prevalence (8.4%), whereas burgers exhibited the lowest (6.3%). These variations suggest that the risk of contamination may be influenced by factors such as food composition, preparation techniques, storage conditions, seasonal timing of sample collection, and hygiene practices during handling and processing [11]. Although *Salmonella* was identified in all types of food samples, the relatively higher occurrence in shawarma, a meat-based product, underscores the need for stricter hygiene protocols during the preparation and handling of such foods.

In Iraq, research specifically targeting RTE foods remains limited. One of the earliest studies in Baghdad investigated 14 types of local and RTE foods, including Kubba, cakes, meats, raw milk, sweets, chicken, olives, vegetables, cheese, cream, eggs, yogurt, butter, fruits, and cold drinks [6]. They reported that *Salmonella* was found in 27% of the samples, with fifteen species and serotypes. Recently, Al-Mashhadany [7] examined grilled chicken meat samples collected from central and suburban retail outlets in Erbil and reported a 7.1% prevalence of *Salmonella*. The identified serovars included *S*. Typhimurium, *S*. Tennessee, *S*. Newport, *S*. Enteritidis, *S*. Anatum, *S*. Arizona, *S*. Muenchen, and S. Montevideo, many of which exhibited antimicrobial resistance. Other studies in Iraq have primarily focused on raw or minimally processed foods. For instance, Zubair et al. [8] reported *Salmonella* contamination in 4.85% of eggshells in Duhok, whereas the egg contents were free from contamination; serovars identified included *S*. Enteritidis, *S*. Typhimurium, and *S*. Typhi. Similarly, Kanaan [9] detected *Salmonella*, predominantly *S*. Enteritidis (63.2%) and *S*. Typhimurium (36.8%), in 12.7% of raw and frozen chicken meat samples in Wasit.

Beyond Iraq, studies in neighboring countries have highlighted *Salmonella* as a significant contaminant in both RTE and raw foods. Mohamed et al. [35] reported the prevalence of *Salmonella* in various foods across Gulf Cooperation Council (GCC) countries. In Saudi Arabia, *Salmonella* was detected in 9.1% of RTE foods, 20% of Shawarma, and 26.7% of Kibtha. In the United Arab Emirates, 46.7% of chicken meat and 1.3% of fresh salad vegetables were contaminated. In Qatar and Kuwait, prevalence in chicken meat reached 16.2% and 1.1%, respectively, while in Bahrain, 39.9% of imported fish were contaminated. More recently, a foodborne outbreak linked to chicken Shawarma in Jordan highlighted gaps in food safety practices during handling and preparation of poultry-based RTE foods [36].

Compared to data from other developing countries, the prevalence of *Salmonella* in RTE foods in Erbil appears to be relatively lower, regardless of the type of food. In a two-year study, of the total 9727 samples from large retailers and canteens in northern Italy, a low rate of *Salmonella* prevalence in RTE (0.07%) foods, where 0.03% in fully cooked items and 0.21% in mixed preparations containing both cooked and raw ingredients was recorded [37]. Similarly, Effimia [38] investigated RTE foods in Athens, Greece, and reported *Salmonella* detection in 1.1% of samples, including sandwiches, cheese and cooked meat with vegetables. On the other hand, slightly higher (6–8%) *Salmonella* prevalence rate was found in different types of meat products in Egypt, closely matching our observed value of 6.3% [39]. In Iran, Jalali et al. [40] examined different types of raw and cooked meat and reported contamination rates ranging from 5.4% to 40%. A recent study reported an exceptionally high contamination rate (52.6%) in grilled smoked artisanal pork sausages in Colombia, with peak values reaching 68.8% in Armenia, particularly among products sold by street vendors [4]. These elevated figures are commonly attributed to cross-contamination, unhygienic preparation environments, lack of refrigeration, inadequate storage, and the use of contaminated raw materials [41,42]. Similarly, in Pakistan, 38% of RTE foods were deemed unfit for human consumption, with 40% of this testing positive for *Salmonella* spp., primarily *S.* Enteritidis (69%) and *S.* Typhimurium (31%) [20]. In India, Singh et al. [43] reported an 8.3% prevalence of *Salmonella* in popular street-vended foods such as panipuri and noodles. In Romania, *Salmonella* was isolated from 4.8% of RTE samples, with *S.* Typhimurium and *S.* Derby being predominant [44]. In contrast, lower prevalence rates are generally reported in developed nations. In the United States, Mamber et al. [45] documented only 0.74% contamination in RTE pork barbecue products from 2005 to 2012. Factors contributing to contamination included cross-contamination, insufficient cleaning, and poor hygiene. Likewise, in Estonia, an overall prevalence of about 1.1% across various food categories, with 0.9% in RTE foods (mainly mayonnaise) and higher rates in raw meat (0.95%) and eggs (2.2%) [46].

In the present study, no statistically significant differences were found in *Salmonella* prevalence based on food types or sources. Although samples from street vendors exhibited a higher (9.2%) contamination rate, this was not statistically significant (*p* > 0.05), suggesting that neither the RTE food type nor the point of sale was a critical determinant of *Salmonella* contamination under the study conditions. Moreover, there is a possibility of Type II error due to small subgroup sizes. This contrasts with some international studies where street-vended foods demonstrated higher contamination risks [4,43].

A notable finding of our study was the seasonal variation in *Salmonella* prevalence, with the highest prevalence observed in June (15.4%) and May (11.5%). These results suggest a seasonal trend where warmer temperatures may enhance the survival, growth, and transmission of *Salmonella* in RTE foods. This seasonal effect is consistent with findings from various regions, including a study in Iran where a higher incidence of *Salmonella* was recorded during the summer months [40], and similar observations from Spain, Switzerland, the Czech Republic and the UK where warmer weather correlated with increased foodborne illness cases [47,48]. These findings reinforce the urgent need for enhanced food safety measures during peak temperature periods to reduce *Salmonella*-related risks.

Furthermore, antibiotic susceptibility testing in this study highlighted important resistance patterns. Overall, the results revealed varying degrees of antimicrobial resistance among the *Salmonella* strains. All *Salmonella* isolates were sensitive to colistin, cefadroxil, and gentamicin. However, high resistance rates were observed for streptomycin (52%) and levofloxacin (48%). Mixed resistance profiles were found for erythromycin, norfloxacin, and kanamycin. These findings are in agreement with reports from India, Pakistan, and Egypt, where an increased multidrug resistance among *Salmonella* strains has been documented [20,49,50].

The present study highlights both the ineffectiveness of certain antimicrobials and the growing concern of antibiotic resistance among *Salmonella* strains recovered from RTE foods. Alarmingly, resistance was observed to streptomycin, levofloxacin, and tetracycline, indicating these antibiotics are no longer effective treatment options for the tested strains, reflecting a multidrug-resistant (MDR) phenotype. These findings are particularly concerning as these antibiotics are commonly used in human and veterinary medicine practices. The resistance patterns observed in this study are consistent with previous findings from India, Pakistan, and Egypt, where MDR *Salmonella* strains have been increasingly reported in foodborne outbreaks and retail food products [20,49,50]. These trends reinforce the urgency of revising empirical treatment guidelines for salmonellosis and call for enhanced antimicrobial stewardship programs.

The seasonal pattern of *Salmonella* prevalence observed in this study increased during warmer months suggesting that elevated ambient temperatures may contribute not only to increased bacterial survival and transmission but also to the spread of resistant strains. Several studies have reported that higher temperatures enhance the growth, survival, and dissemination of *Salmonella* along the food production and distribution chain [47,48]. A systematic review and meta-analysis confirmed that foodborne *Salmonella* infections increased significantly with ambient temperatures, particularly during summer [51]. Moreover, recent studies have shown that environmental factors, including temperature, can influence the persistence and transfer of antimicrobial resistance genes in bacterial populations [52]. This seasonal dynamic further emphasizes the need for proactive food safety interventions during summer. Moreover, developing and/or improving regulations on the use of antibiotics in food-producing animals is crucial to prevent the dissemination of resistance from farm to fork, ensuring both food safety and public health.

## 5. Conclusions

This study reveals a moderate but significant prevalence of *Salmonella* in ready-to-eat (RTE) foods in Erbil, with notable seasonal variations and concerning trends in antimicrobial resistance. The higher detection rates during warmer months underscore the influence of temperature on microbial proliferation in RTE foods. Although the overall contamination level was relatively lower (7.1%) compared to other reported studies (ranging from 40% to 69%), the presence of resistant strains remains a pressing public health issue. The study results would help in source tracking of *Salmonella*, whereas antimicrobial resistance profiles can guide empirical treatment during outbreaks. Educating communities on handwashing, safe food handling, and water treatment can reduce transmission and mitigate health risk. These findings call for an integrated “One Health” approach addressing human, animal, and environmental health collectively to combat *Salmonella* transmission and antimicrobial resistance. Strengthening food safety regulations, improving hygiene practices in food preparation and vending, and enforcing responsible antibiotic usage in agriculture are essential strategies. Finally, public awareness campaigns on safe food handling, storage, and consumption practices can help mitigate the burden of foodborne infections. Based on these results, further research is warranted to investigate strain diversity and resistance mechanisms using genomics tools to better understand the strain diversity.

## Figures and Tables

**Table 1:** Distribution of *Salmonella* contamination in RTE food samples, categorized by food type and source.

Source	Total Number of Samples	Total Positive Samples (%)	Positive Samples/Total Number of Samples (%)			
			Sandwiches	Pastries	Shawarma	Burgers
Cafeteria	115	8 (6.9)	2/28 (7.1)	1/25 (4.0)	3/30 (10.0)	2/32 (6.3)
Street foods	120	11 (9.2)	2/30 (6.7)	2/25 (8.0)	4/35 (11.4)	3/30 (10.0)
Restaurants	115	6 (5.2)	2//27 (7.4)	2/25 (8.0)	1/30 (3.3)	1/33 (3.1)
Total	350	25 (7.1)	6/85 (7.1)	5/75 (6.7)	8/95 (8.4)	6/95 (6.3)

**Table 2:** Contamination rates and 95% confidence intervals (CI) of *Salmonella* spp. in ready-to-eat (RTE) foods from cafeterias, street vendors, and restaurants.

Food Type (n)	Source (n)	Positive/Total (%)	95% CI (%) *	Total Contamination (%)	95% CI (%) *
Sandwiches (85)	Cafeterias (28)	2/28 (7.1)	2.0–22.6	6/85 (7.1)	4.0–12.9
	Street vendors (30)	2/30 (6.7)	1.8–21.3		
	Restaurants (27)	2/27 (7.4)	2.1–23.4		
Pastries (75)	Cafeterias (25)	1/25 (4.0)	0.7–19.5	5/75 (6.7)	2.8–14.3
	Street vendors (25)	2/25 (8.0)	2.2–25.0		
	Restaurants (25)	2/25 (8.0)	2.2–25.0		
Shawarma (95)	Cafeterias (30)	3/30 (10.0)	3.5–25.6	8/95 (8.4)	4.3–15.7
	Street vendors (35)	4/35 (11.4)	4.5–25.3		
	Restaurants (30)	1/30 (3.3)	0.6–16.7		
Burgers (95)	Cafeterias (32)	2/32 (6.3)	1.7–20.1	6/95 (6.3)	2.9–13.1
	Street vendors (30)	3/30 (10.0)	3.5–25.6		
	Restaurants (33)	1/33 (3.1)	0.6–15.8		
Total (350)	All sources	25/350 (7.1)	4.9–10.3	25/350 (7.1)	4.9–10.3

n: number of samples; *: no statistically significant differences (*p* > 0.05) were observed in *Salmonella* contamination rates between food types or sources.

**Table 3:** Antimicrobial susceptibility patterns of *Salmonella* strains grouped by antimicrobial class.

Class	Antimicrobial Agent	Sensitive n (%)	Intermediate n (%)	Resistant n (%)
Polymyxins	Colistin (CST10)	25 (100)	0 (0)	0 (0)
Macrolides	Erythromycin (ERY15)	11 (44)	5 (20)	9 (36)
Cephalosporins	Cefadroxil (CFR30) (1st gen.)	25 (100)	0 (0)	0 (0)
	Cefotaxime (CTX30) * (3rd gen.)	23 (92)	2 (8)	0 (0)
Aminoglycosides	Neomycin (NEO30)	12 (48)	4 (16)	9 (36)
	Gentamicin (GM15) *	25 (100)	0 (0)	0 (0)
	Kanamycin (KAN30)	14 (56)	3 (12)	8 (32)
	Streptomycin (STR10)	9 (36)	3 (12)	13 (52)
	Tobramycin (TM10) *	22 (88)	0 (0)	3 (12)
Fluoroquinolones	Norfloxacin (NOR10)	10 (40)	7 (28)	8 (32)
	Levofloxacin (LEV5) *	8 (32)	5 (20)	12 (48)
Tetracyclines	Tetracycline (TEC30)	14 (56)	2 (8)	9 (36)

n: number of samples; *: Agents included under CLSI (2023) guidelines. gen.: generation.

**Table 4:** Temporal distribution of *Salmonella* in RTE food samples.

Month	Total Samples	Total Positive Samples (%)	Sandwiches n (%)	Pastries n (%)	Shawarma n (%)	Burgers n (%)
January	58	1 (1.7)	0/14 (0)	0/9 (0)	1/16 (6.3)	0/19 (0)
February	59	1 (1.8)	0/14 (0)	0/8 (0)	0/23 (0)	1/14 (7.1)
March	63	3 (4.8)	1/14 (7.1)	1/15 (6.7)	1/15 (6.7)	0/19 (0)
April	57	5 (8.8)	1/15 (6.7)	1/13 (7.7)	2/14 (14.3)	1/15 (6.7)
May	61	7 (11.5)	2/15 (13.3)	2/16 (12.5)	1/15 (6.7)	2/15 (13.3)
June	52	8 (15.4)	2/13 (15.4)	1/14 (7.1)	3/12 (25)	2/13 (15.4)
Total	350	25 (7.1)	6/85 (7.1)	5/75 (6.7)	8/95 (8.4)	6/95 (6.3)

n: number of samples.

## Data Availability

The data presented in this study are available on request from the corresponding author. The data are not publicly available, since the respective data is part of an ongoing study.
